# Footprint Curvature in Spanish Women: Implications for Footwear Fit

**DOI:** 10.3390/ijerph17061876

**Published:** 2020-03-13

**Authors:** Carolina Alonso-Montero, Anselén Torres-Rubio, Nuria Padrós-Flores, Emmanuel Navarro-Flores, José Vicente Segura-Heras

**Affiliations:** 1 Departamento de Patología y Cirugía. Universidad Miguel Hernández de Elche. Crta. N 332 Km 87 s/n., 03550 Sant Joan d’Alacant (Alicante), Spain; c.alonso@umh.es (C.A.-M.); anselen.torresr@umh.es (A.T.-R.); 2 Departamento de Ciencias de Comportamiento y Salud. Universidad Miguel Hernández de Elche. Crta. N 332 Km 87 s/n., 03550 Sant Joan d’Alacant (Alicante), Spain; npadros@umh.es; 3 Frailty and cognitive impairment organized group (FROG), University of Valencia, 46010 Valencia, Spain; 4 Department of Nursing, University of Valencia, c/Jaume Roig s/n, 4610 Valencia, Spain; 5 Instituto Centro de Investigación Operativa, Universidad Miguel Hernández de Elche. Crta. N 332 Km 8 s/n., 03550 Sant Joan d’Alacant (Alicante), Spain; jvsh@umh.es

**Keywords:** shoes, anthropometry, foot diseases, clustering

## Abstract

The incorrect adjustment of footwear produces alterations in the foot that affect quality of life. The usual measurements for shoe design are lengths, widths and girths, but these measures are insufficient. The foot presents an angle between the forefoot and the rearfoot in the transverse plane, which is associated with foot pronation, hallux valgus and metatarsus adductus. Here, we aimed at identifying the groups formed by the angulations between the forefoot and rearfoot using a sample of footprints from 102 Spanish women. The angle between the forefoot and rearfoot was measured according to the method described by Bunch. A cluster analysis was performed using the K-means algorithm. Footprints were grouped into three types: curved, semi-curved and straight, according to the degrees of angulation between the forefoot and rearfoot. There is great variability in the morphology of the foot. Based on our findings, to achieve a better footwear fit, we propose the manufacture of three types of lasts with different curvatures.

## 1. Introduction

Footwear can cause foot injuries, sometimes due to incorrect fit [1,2,3], which causes foot health problems, reduces quality of life and in some cases can cause injuries in diabetic foot, due to the use of inappropriate footwear [4,5,6,7,8] Dufour et al. [1] reported a statistically significant association in women between rearfoot pain and having used incorrect footwear in the past. Menz et al. [9] observed that women who had used narrow footwear between the ages of 20 and 39 years more frequently developed hallux valgus. Hurst et al. [10] showed that with changes in the design of the toe of the footwear and in the material of the upper, the pressures to which the toes are subjected can be reduced. 

Kouchi [11] suggested that to reduce the pressure on the lateral edge of the forefoot, it is necessary to change the curvature of the latter [12]. Calvo-Lobo et al. [13] evaluated the length and width to assess the fit of footwear taking into account the studies of Kouchi [11]. To achieve a good fit, it is necessary to assess the curvature of the footwear, especially since those with Down syndrome are mostly flatfooted.

Shoe fit is a complex issue that is influenced by multiple factors. There are differences in the ratio between the length and width of the foot by age [14,15], sex [16] and ethnicity [17,18,19,20], and even within the same sex and ethnicity [21,22,23]. To these factors should be added that the foot is a mobile structure that changes shape during different phases of gait [24], with a flattening of the foot during the mid-stance and elongation of the front of the rearfoot after forefoot contact [25]. Furthermore, not all feet move in the same way during the gait. Levinger et al. [26] observed differences in the movement of the foot during walking in subjects with a normal internal arch and subjects with a flattened internal arch, with the latter group presenting greater abduction of the forefoot with respect to the rearfoot in the mid-stance phase of the gait. 

Buldt et al. [27,28] identified differences in the forefoot–rearfoot relationship in the transverse plane when comparing gait between pronate, normal and flat feet. Redmond [29] connected forefoot abduction with a pronated foot, and forefoot adduction with a supinated foot. A similar result was reported by Lee et al. [30], who related the degree of abduction of the forefoot with the movement caused by the internal or external rotation of the tibia, although their findings should be considered with caution since they were based on a small sample (just six subjects). For this reason, it is common to use shoes with different curvatures in the transverse plane, depending on the type of foot [29,31].

Although it is clear that the shoe last is not intended to be a reproduction of the foot, and it has clear differences [32], it should not be forgotten that the footwear should adapt to foot morphology during the gait. Traditionally, in the design of last lengths, the widths and girths of feet have been used [23]. Some authors [3,11,33,34] have suggested that these measures are not enough for a proper shoe fit. Hill et. al. [22] assessed the differences in the height of the arch in relation to the length of the foot for the sizing of the lasts, finding that there is no relationship between the length of the foot and the height of the arch. Lee et al. [30] found changes in the length of the foot on the medial or lateral side, which would have effects on footwear design.

The position of the forefoot relative to the rearfoot produces an angle or curve in the foot that is also reflected on the last. Kouchi and Tsutsumi [12], studying the outline of the foot, found greater pronation and outflare in the feet of their sample than in the usual shoe lasts. Goonetilleke and Luximon [33] determined that the curve in the foot is not related to the width or length of the foot. Later, Luximon and Goonetilleke [34] created a predictive model for shoe adjustment, from which it was deduced that the weight of the mould’s curvature is greater than the weight of its width.

Several authors have assessed the forefoot–rearfoot ratio in the transverse plane. Bunch [3] reported measures intended to improve the feet of athletic shoes for women. Kouchi [12] concluded that pronation is the factor that most influences the shape of the foot, and most of the subjects in her sample had a foot with less angulation than the lasts. Tsutsumi [12] suggested the creation of two types of lasts—the standard or usual last, which is already manufactured, and another last for those subjects with higher pronation. Goonetilleke and Luximon [33] created a new method in which the calculation of the central point or origin of angulation is included. Rodrigo et al. [35] identified four foot types based on foot outline. Domínguez and Munuera [36] used radiographic images in dorsoplantar projection to obtain the mean values of the metatarsus adductus angle.

Although different methods can be found, results are not comparable between them; all authors have agreed that the angulations of the last and the foot do not coincide, which could be associated with shoe fitting problems.

This study aimed to determine the values of the angle between the forefoot and rearfoot in the transverse plane in a sample of Spanish women, which allows the grouping of footprints. This allows us to classify different types of feet according to their angles within the same shoe size, with corresponding implications for the design and fit of the shoe. The hypothesis of this research was that it is possible to design and classify feet according to their angles within the same shoe size.

## 2. Materials and Methods 

### 2.1. Participants

The sample consisted of 102 women between the ages of 18 and 45 years who reported regular use of a 38 shoe size (according to the European numbering); this shoe size was chosen because it is typically used during fit testing in footwear manufacturing [37]. The study included those women who do not use orthosis in the lower limb or special shoes, who are asymptomatic and who have not undergone surgery on the foot and/or ankle. All subjects provided informed consent for inclusion in the study before participating. The study was conducted in accordance with the Declaration of Helsinki [38]. The study was approved by the Ethics Committee of Miguel Hernandez University (2016.45.E.OEP; 2016.108.E.OEP). The sample was homogenised to prevent variations associated with sex, age and foot size.

### 2.2. Instrumentation and Procedure

The footprint of a single foot was collected to avoid duplicates in the sample, as recommended by Menz [39]—in this case the right foot, as indicated by Kouchi and Tsutsumi [12] and Rodrigo [35]. The footprint of each participant was obtained using a foot imprinter. The subjects were standing, distributing their weight on both feet and relaxed, and thus maintaining their usual base and angle of gait.

**Figure 1:**
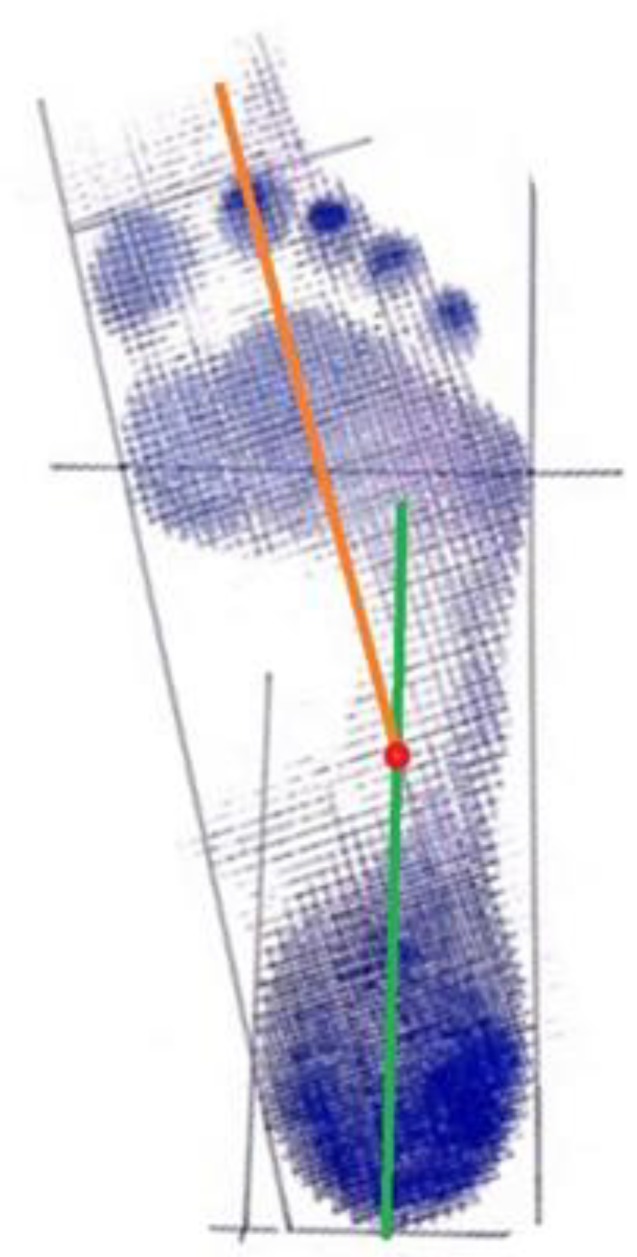
Footprint with measurements according to the method described by Bunch.

The method described by Bunch [3] was used to measure the angle between the forefoot and hindfoot (Figure 1).

The footprint length was measured along the longitudinal axis (axis running through the centre of the heel and half the width of the forefoot), from the most proximal aspect of the heel to the most distal point of the longest toe. The measurements were made in millimetres by a single researcher.

All measurements were taken three times for each footprint, and the standard deviation (<2%) was calculated. The average of the three measurements was used.

### 2.3. Statistical Analysis

The data were analysed with IBM^®^ SPSS^®^ v23 software (IBM Corp., Armonk, N.Y., USA). Descriptive statistics were obtained and a cluster analysis was performed using the K-means algorithm. 

## 3. Results

### 3.1. Participant Characteristics

The mean age of the study subjects was 29.13 ± 7.99 years. All subjects reported using a size 38 shoe, and the average footprint length was 231.63 ± 5.46 mm. All subjects used a shoe of the same length, but there was a 24 mm difference between the shortest and the longest foot (Table 1).

We obtained an average angle between the forefoot and rearfoot of 12.00° with a standard deviation of 3.34° using the Bunch method [3].

### 3.2. Cluster Analysis

**Figure 2:**
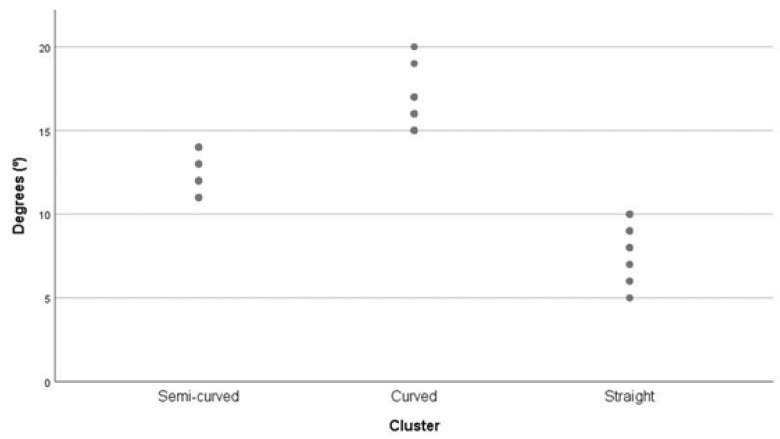
Cluster analysis for forefoot–rearfoot angulation.

Cluster analysis was performed using the K-means algorithm for the angle between the forefoot and rearfoot. We obtained three groups (Figure 2). Table 2 shows the final clusters’ centroids, together with the associated dispersion and the number of footprints grouped in each cluster.

Table 2 shows the final clusters’ centroids, together with the dispersion associated with them and the number of footprints grouped in each cluster.

## 4. Discussion

The purpose of this study was to determine the values of the angle between the forefoot and rearfoot in the transverse plane in a sample of Spanish women, which allowed the grouping of footprints.

To avoid introducing unnecessary confounding factors into the analysis, we selected a sample of women from the same population using the same shoe size [16,18,19,21,22,40,41]. However, among the women in the sample, there was a 24 mm difference between the longest and the shortest footprint, indicating that not all subjects have the same shoe fit.

The use of shoes with an incorrect length can cause foot injuries [5,9,13,42,43]. Menz et al. [44] observed, in a geriatric population, that when looking for an adequate adjustment to the width of the foot, shoes longer than the usual recommendations were chosen. The use of excessively long footwear causes an increase in the muscular load, movement is less efficient and the feeling of walking deteriorates [45]. Sometimes, the problem on the side of the forefoot, especially in the V toe, is due to the curvature of the last, rather than to the width in that area [11].

Data cannot be compared with Bunch’s work [3] because their design of the new last does not provide data on the values obtained in the measurements.

The angle obtained between the forefoot and rearfoot reflects the ab-adduction of the forefoot relative to the rearfoot in the transverse plane. Most abduction of the forefoot in relation to the rearfoot is associated with increased foot pronation [11,26,29,46]. This pronation is associated with a lower arch height [11,12,26], which has traditionally served to classify different types of feet. Buldt et al. [27,28] observed differences in movement between normal, cavus and flat feet. A similar result was found by Lee et al. [30], who related the degree of ab-adduction of the forefoot with the internal or external rotation of the tibia motion.

Large inter-subject variability was observed, with footprints being classified into three groups depending on the forefoot–rearfoot angle. The manufacture of lasts with different curvatures could greatly improve shoe fit, avoiding compression on the lateral side of the forefoot.

These results are similar to those obtained by Rodrigo et al. [35], who, based on the study of the foot outline at 2 and 40 mm from the floor, identified four types of feet depending on the medial and lateral side of the foot outline. The results of Rodrigo et al. were similar to those obtained by Kouchi and Tsutsumi [12], who proposed the production of a last for a pronated foot, with angulation in the back of the last, while maintaining the usual last for those subjects who do not present angulation in the back of the foot.

In a study published in 1995, Kouchi [11] considered that in order to adjust the curve of the last to the foot, it would be necessary to change the design of the back part of the last since the foot is pronated and outflared in most people, whereas the lasts are straight, designed for feet without pronation or outflare. This causes pressure in the forefoot, especially on the fifth toe, and the simple change of the toe does not relieve the pressure in this area.

In podiatry, recommendations regarding the angulation of the last are common according to the type of foot. Barton et al. [31] recommended the use of straight footwear for pronated feet and curved footwear for supinated feet, while Redmond [29] recommended curved lasts for supinated feet. The possibility of having lasts with different degrees of curvature, closer to the actual population’s measurements, would improve the fit of footwear and, ultimately, foot health.

The use of different measurement methods makes it difficult to compare results between different works. If the forefoot–rearfoot ratio is influenced by the position of the subtalar joint, the position of the subject when the data is collected might modify the values obtained. There are studies with subjects in different positions, such as standing, sitting down and standing with a relaxed position. It is necessary to provide the position of the subject for data collection in different studies.

The use of the heel also modifies the position of the subtalar joint, and different values could be obtained from those acquired with the foot on a flat surface.

On the other hand, differences in the morphology of the foot have been documented according to foot size, sex, ethnicity and age, so it is necessary to expand the research with other samples.

## 5. Conclusions

There is great variability in the morphology of the foot, and it is difficult for a single form to adjust to so much variability. We could group footprints into three types depending on the angle between the forefoot and rearfoot, thereby improving shoe fit and foot health.

The forefoot–rearfoot relationship depends on the position of the foot. It would be interesting to evaluate studies of foot position to test its influence on foot curvature, and to record foot position during data collection.

## Figures and Tables

**Table 1:** Statistical summary (N = 102).

Variable	Mean (SD)	Minimum	Maximum
Age (years)	29.13 (7.99)	18	45
Foot length (mm)	231.63 (5.46)	220	244
Forefoot–rearfoot angle (degrees)	12.00 (3.34)	5	20

Abbreviations: SD, standard deviation; mm, millimetres.

**Table 2:** Final cluster centroids.

Type of Angulation	N	Forefoot–Rearfoot Angle Mean (SD)
Curved	29	16.03° (1.24°)
Semi-curved	35	12.49° (1.12°)
Straight	38	8.47° (1.56°)
